# Nanocarrier-based delivery of siRNA therapeutics in rheumatoid arthritis: immune mechanisms and translational perspectives

**DOI:** 10.3389/fimmu.2025.1718256

**Published:** 2025-12-17

**Authors:** Longbin Bai, Peng Su

**Affiliations:** Department of Hand and Foot Surgery, Shandong Provincial Hospital Affiliated to Shandong First Medical University, Jinan, China

**Keywords:** nanocarriers, rheumatoid arthritis, RNA interference, siRNA, targeted therapy

## Abstract

Rheumatoid arthritis (RA) is a chronic autoimmune disease characterized by persistent synovial inflammation, pannus formation, and progressive joint destruction. Conventional therapies, including methotrexate, NSAIDs, and biologics, have improved outcomes but remain limited by incomplete efficacy, adverse effects, and resistance. Small interfering RNA (siRNA) has emerged as a promising strategy due to its ability to selectively silence pathogenic genes such as TNF-α, IL-1β, IL-6, IL-17, VEGFA, and key signaling pathways including NF-κB, JAK/STAT, and MAPK. Preclinical studies have shown that siRNA can suppress inflammation, reduce pannus formation, and protect cartilage; however, clinical translation is hindered by instability, nuclease degradation, poor biodistribution, and off-target effects. Nanocarrier-based systems offer solutions by improving siRNA stability, cellular uptake, and targeted delivery to inflamed joints. Lipid nanoparticles, PLGA, chitosan, and polyethyleneimine have been widely studied, while emerging carriers such as dendrimers, self-assembling peptides, mesoporous silica, and metal-organic frameworks (MOFs) further enhance controlled release and specificity. Functional modifications with ligands such as folic acid, hyaluronic acid, or RGD peptides enable active targeting, and stimuli-responsive designs allow pH-, ROS-, or enzyme-triggered release. Theranostic platforms also provide opportunities for real-time monitoring of biodistribution and therapeutic efficacy. Overall, siRNA-based nanomedicine represents a promising therapeutic paradigm for rheumatoid arthritis; however, its clinical translation remains constrained by several important challenges. Although current nanocarrier platforms demonstrate strong gene-silencing efficiency and encouraging anti-inflammatory outcomes in preclinical models, their behavior in humans is far less predictable. Key obstacles—including systemic stability, protein corona formation, endosomal escape efficiency, batch-to-batch manufacturing consistency, and long-term biosafety—must be rigorously addressed before clinical application can be realized. In addition, the heterogeneous nature of RA and its fluctuating inflammatory microenvironment imply that a single siRNA target or delivery strategy may not be universally effective across patient populations. Regulatory considerations also pose significant barriers, as siRNA nanomedicines must meet strict requirements for GMP production, quality control, sterility, pharmacokinetics, immunogenicity, and degradation profiling.

## 1 Introduction

Rheumatoid arthritis (RA) is a chronic systemic autoimmune disease characterized by persistent synovial inflammation, pannus formation, and progressive joint destruction. The pathogenesis of RA involves complex interactions between innate and adaptive immune responses, pro-inflammatory cytokine networks, and aberrant activation of intracellular signaling pathways, ultimately leading to irreversible cartilage and bone damage (1, 2). Despite advances in pharmacotherapy over the past decades, RA remains a major cause of disability and reduced quality of life worldwide (3).

Conventional therapeutic strategies, including methotrexate (MTX), nonsteroidal anti-inflammatory drugs (NSAIDs), and biologic agents targeting cytokines or immune cells, have greatly improved disease outcomes (4). However, these agents are often limited by incomplete efficacy, systemic adverse effects, high costs, and the emergence of drug resistance (5). These limitations highlight the unmet need for novel therapeutic approaches that can selectively and sustainably modulate disease-driving molecular mechanisms.

Nevertheless, the clinical translation of siRNA therapeutics faces significant challenges. Naked siRNAs are inherently unstable in biological fluids, prone to rapid enzymatic degradation, and exhibit poor cellular uptake and suboptimal biodistribution (6). Moreover, systemic administration can trigger off-target effects and unintended immune responses (7). These barriers underscore the critical need for effective delivery platforms capable of protecting siRNA, enhancing cellular internalization, and achieving site-specific release within the inflamed synovium.

Rheumatoid arthritis is a fundamentally multifactorial disorder driven by the interplay of numerous cytokines, multiple pathogenic cell subsets, and a constellation of intracellular signaling pathways including NF-κB, JAK/STAT, MAPK, and PI3K/Akt (1). Unlike small-molecule drugs or biologics that typically inhibit a single receptor or cytokine, siRNA therapeutics operate at the post-transcriptional level, enabling precise silencing of virtually any gene implicated in RA pathogenesis (6). This makes siRNA uniquely suited for a disease in which targeting a single cytokine or pathway often provides incomplete or transient benefit. Furthermore, multiple siRNA sequences can be combined within a single formulation, allowing simultaneous suppression of converging inflammatory mediators—such as TNF-α, IL-1β, or STAT3—to disrupt entire pathogenic circuits rather than isolated nodes (8). siRNA also provides the flexibility to modulate intracellular factors that are undruggable by conventional therapeutics, including transcription factors, adaptor proteins, or intracellular kinases that play central roles in synovial hyperplasia and immune dysregulation. Importantly, by selectively reprogramming macrophage polarization or fibroblast phenotypes, siRNA can reshape the RA microenvironment at a systems-level scale, addressing both inflammatory and structural components of disease. Together, these features position siRNA as a uniquely powerful modality for confronting the molecular complexity and cellular heterogeneity that define rheumatoid arthritis.

Nanocarrier-based drug delivery systems offer a promising solution to these challenges. Engineered nanomaterials—including lipid nanoparticles, polymeric nanocarriers, and inorganic nanoparticles—have shown the ability to encapsulate siRNA, protect it from degradation, and facilitate targeted delivery to diseased tissues (9–11). Furthermore, advanced designs incorporating pH-, enzyme-, or reactive oxygen species (ROS)-responsive elements enable controlled and stimuli-sensitive release within the inflammatory microenvironment of RA (12). These innovations not only improve therapeutic efficacy but also minimize systemic toxicity, paving the way for the application of siRNA-based precision medicine in RA management.

Though several reviews have discussed siRNA therapeutics or nanocarrier platforms independently, an integrated analysis linking nanocarrier design principles to the immunopathology of rheumatoid arthritis is still lacking. Moreover, recent advances in dual-responsive polymeric systems, metal–organic framework (MOF)-based co-delivery platforms, macrophage-reprogramming nanocarriers, and lipid–polymer hybrid architectures have not been synthesized in previous literature. To address this gap, the present review provides a comprehensive and updated framework that connects nanomaterial engineering with immune-regulatory mechanisms, including cytokine modulation, macrophage polarization, angiogenesis, and intracellular signaling pathways. In addition, we incorporate a translational perspective, discussing protein corona formation, complement activation–related immunotoxicity, GMP manufacturing barriers, pharmacokinetic constraints, and regulatory challenges that must be overcome for successful clinical translation. This multidimensional approach highlights the unique contribution of our review and underscores its relevance for advancing siRNA-based nanomedicine in RA.

## 2 Pathogenesis of RA and potential siRNA targets

### 2.1 Cytokine networks in rheumatoid arthritis and siRNA targets

Cytokines are pivotal drivers of the inflammatory milieu in rheumatoid arthritis (RA). Pro-inflammatory mediators such as TNF-α, IL-1β, IL-6, and IL-17 are markedly elevated in synovial tissue, where they sustain chronic inflammation and mediate joint destruction (13). TNF-α acts as a central regulator, stimulating synovial fibroblasts, endothelial cells, and macrophages, thereby promoting pannus formation and osteoclast differentiation. siRNA targeting TNF-α has demonstrated efficacy in reducing synovial inflammation and cartilage erosion in preclinical arthritis models (8). IL-6 contributes to systemic and local inflammation by supporting B-cell differentiation, Th17 expansion, and osteoclast activation, and siRNA against IL-6 or its receptor effectively decreases inflammatory burden (14). IL-17, predominantly produced by Th17 cells, amplifies inflammation by inducing secondary cytokines such as IL-6 and GM-CSF, along with matrix metalloproteinases (MMPs) that degrade cartilage (15). Silencing IL-17 expression via siRNA has been shown to suppress joint swelling and cartilage degradation. Beyond classical cytokines, VEGFA—primarily known for its angiogenic role—also promotes synovial neovascularization and immune cell infiltration, exacerbating inflammation (16). siVEGFA reduces pannus vascularization and inflammatory infiltration, underscoring the therapeutic potential of targeting angiogenic cytokines in RA. Collectively, these findings highlight cytokine-targeted siRNAs as a promising approach to interrupt the self-perpetuating inflammatory network in RA.

It is also important to recognize that optimal siRNA target selection may vary across different stages of RA, reflecting the evolving molecular landscape of the disease. In early RA, the synovium is dominated by acute inflammatory activity characterized by high levels of TNF-α, IL-1β, IL-6, and IL-17, accompanied by dense infiltration of macrophages, neutrophils, and Th17 cells. At this stage, siRNAs directed against pro-inflammatory cytokines or upstream regulators such as NF-κB, JAK2, or STAT3 may be particularly effective in dampening cytokine cascades and preventing irreversible synovial remodeling. In contrast, late-stage RA exhibits a very different pathological signature, including fibroblast-driven pannus expansion, increased extracellular matrix density, hypoxia-induced angiogenesis, and enhanced osteoclastogenesis that drives bone erosion (17). Consequently, siRNAs targeting fibroblast-associated molecules, angiogenic mediators, matrix-degrading enzymes, or osteoclastogenic regulators may provide greater therapeutic benefit in chronic disease (18). These stage-dependent differences highlight that no single siRNA target is universally optimal across the RA continuum, underscoring the need for timing-specific or combination siRNA strategies tailored to the dynamic immunopathology of the disease.

### 2.2 Aberrant signaling pathways in RA and siRNA targets

In addition to extracellular cytokine networks, multiple intracellular signaling pathways are aberrantly activated in RA, driving sustained inflammation and structural damage (19). The NF-κB pathway is a central mediator of synovial inflammation, regulating the transcription of TNF-α, IL-1β, and chemokines. Persistent NF-κB activation in macrophages and fibroblast-like synoviocytes (FLS) promotes hyperplasia and osteoclastogenesis, while siRNAs targeting the NF-κB pathway inhibit phosphorylation of IκBα and nuclear translocation of p65, thereby reducing transcription of pro-inflammatory cytokines such as TNF-α and IL-1β. For example, Duan et al. demonstrated that NF-κB p65 siRNA delivered via a folate-modified lipid nanoparticle markedly downregulated iNOS and COX-2 expression, leading to decreased macrophage activation and cartilage protection in CIA mice (20). In the JAK/STAT axis, siRNA-mediated silencing of JAK2 or STAT3 blocks downstream phosphorylation and nuclear localization of STAT3, attenuating IL-6- and IL-23-driven Th17 differentiation and inflammatory cytokine production. Liu et al. further confirmed that suppression of p-STAT3 by JAK2 siRNA results in upregulation of anti-inflammatory regulators such as SOCS3 and BCL2L1, contributing to immune homeostasis (21, 22). The MAPK cascade (p38, ERK, JNK), triggered by TNF-α and IL-1β, drives MMP production and cartilage degradation; silencing p38 MAPK with siRNA attenuates MMP expression and protects cartilage (23–25). Dysregulation of RANK/RANKL signaling plays a critical role in osteoclastogenesis and bone erosion, while siRNA against RANKL or NFATc1 has shown efficacy in suppressing osteoclast differentiation (26). Furthermore, the PI3K/Akt/mTOR pathway supports FLS proliferation, angiogenesis, and survival, exacerbating synovial pathology (27). siRNA-mediated inhibition of mTOR has been demonstrated to reduce synovial angiogenesis and partially restore immune homeostasis (28). Additionally, TLR-related pathways, which initiate innate immune responses via MyD88-dependent signaling, have been effectively modulated by siRNAs targeting TLR4 and MyD88. Such interventions suppress downstream NF-κB and MAPK activation, reducing TNF-α, IL-6, and IL-12 secretion from macrophages and dendritic cells (29). Together, these studies highlight the central role of intracellular signaling cascades in RA pathogenesis and underscore the potential of siRNA-based therapies to precisely silence key molecular drivers.

Moreover, siRNA delivery systems can modulate the immune microenvironment by influencing macrophage polarization (30). In RA, pro-inflammatory M1 macrophages dominate the synovial tissue, producing TNF-α, IL-1β, and ROS that perpetuate joint inflammation and matrix degradation (31). Conversely, M2 macrophages exhibit anti-inflammatory and tissue-repair functions through the release of IL-10 and TGF-β. Targeted siRNAs—particularly those against NF-κB p65, JAK2/STAT3, or SOCS1—have been shown to inhibit M1-related signaling while promoting M2 polarization (32). This shift from an M1- to M2-dominant phenotype reduces synovial inflammation, enhances debris clearance, and facilitates cartilage repair. Nanocarrier-based siRNA formulations, such as hyaluronic acid-modified or ROS-responsive nanoparticles, further amplify this effect by preferentially accumulating in activated macrophages within inflamed joints and ensuring controlled intracellular release (33).

Collectively, these findings underscore that siRNA therapeutics exert multi-layered immunomodulatory effects—not only by directly silencing pro-inflammatory signaling molecules such as NF-κB, JAK2, and STAT3, but also by reprogramming the immune landscape of the RA microenvironment through macrophage repolarization and transcriptional re-balancing of downstream targets. Such dual regulation of signaling and cellular phenotype represents a promising direction for the next generation of RNAi-based nanotherapeutics in RA.

## 3 RNA interference and its application in rheumatoid arthritis

In August 2018, Patisiran became the world’s first RNA interference (RNAi)–based therapeutic approved in the United States and Europe, marking the advent of a new era in clinical medicine (34). This milestone brought RNAi from preclinical discovery into the clinical spotlight. While gene therapy was traditionally defined as interventions targeting genomic DNA, such as CRISPR-Cas9–based genome editing, RNAi represents an alternative post-transcriptional regulatory strategy, expanding the scope of gene-based therapeutics (35–37).

RNAi is an evolutionarily conserved mechanism of gene silencing mediated by small RNA molecules. Broadly, RNAi encompasses microRNA (miRNA) and small interfering RNA (siRNA) pathways. miRNAs are endogenous single-stranded RNAs that bind partially complementary sequences on target mRNAs, thereby regulating gene expression by degrading mRNA, reducing its stability, or inhibiting translation (38). In contrast, siRNAs are typically 20–25 nucleotide double-stranded RNAs with perfect complementarity to their target mRNA, resulting in direct cleavage and degradation of the transcript. siRNAs can be either endogenous products of precursor transcripts or exogenously synthesized duplexes.

**Figure 1:**
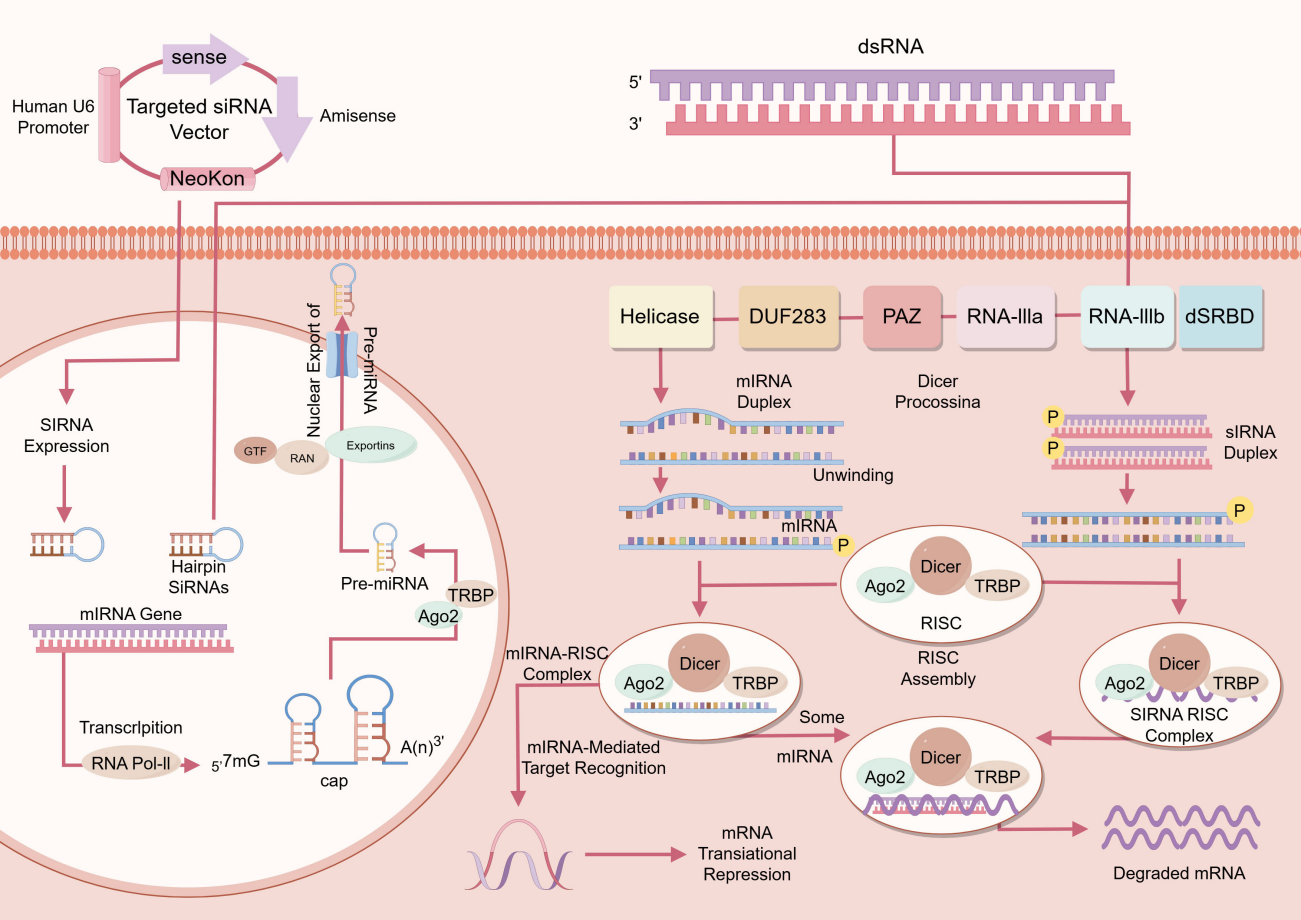
Schematic diagram of RNA interference (RNAi). This figure illustrates the process of small interfering RNA (siRNA)-mediated gene silencing, which involves several key steps. siRNAs can be introduced into cells either through expression vectors (e.g., driven by a U6 promoter) or as chemically synthesized double-stranded RNA (dsRNA). Inside the cell, expression vectors produce hairpin siRNAs, which are processed into siRNA precursors, whereas endogenous microRNAs (miRNAs) are transcribed as pri-miRNAs, cleaved into pre-miRNAs, and exported from the nucleus by Exportin proteins. In the cytoplasm, Dicer, together with its functional domains (PAZ, RNAse IIIa/IIIb, dSRBD) and cofactors such as TAR RNA-binding protein (TRBP) and Argonaute 2 (Ago2), processes dsRNA or pre-miRNA into 20–25 nucleotide siRNA duplexes. These duplexes are subsequently unwound, and only the guide strand is incorporated into the RNA-induced silencing complex (RISC), while the passenger strand is degraded. The assembled siRNA-RISC complex recognizes target mRNAs through complementary base pairing. When perfect complementarity is achieved, Ago2 cleaves the target mRNA, leading to its degradation and transcriptional silencing; in the case of miRNAs with partial complementarity, translational repression or destabilization of mRNA predominates. Ultimately, siRNA-mediated RNA interference results in the specific silencing of target genes at the post-transcriptional level, forming the theoretical basis for therapeutic applications in diseases such as rheumatoid arthritis, where selective knockdown of inflammatory cytokines or signaling molecules can alleviate pathological processes.

The canonical RNAi process involves three stages (**Figure 1**): (i) initiation, in which siRNA precursors are processed by Dicer and exported into the cytoplasm; (ii) effector formation, in which siRNAs are incorporated into the RNA-induced silencing complex (RISC), followed by unwinding and guide-strand selection to target complementary mRNA for degradation by Argonaute 2 in an ATP-dependent manner; and (iii) amplification, in which secondary siRNA synthesis reinforces the silencing cascade, thereby ensuring robust suppression of the target gene.

The therapeutic potential of RNAi in rheumatoid arthritis (RA) has been demonstrated in numerous preclinical studies. Early proof-of-concept experiments employed electroporation-mediated siRNA transfection into joint tissues, successfully silencing TNF-α and alleviating synovial inflammation (39). Since then, a wide range of siRNAs targeting key inflammatory cytokines (e.g., TNF-α, IL-1β, IL-6) or pathogenic pathways (e.g., NF-κB, JAK/STAT) have been designed to suppress inflammation and attenuate joint destruction (40–43). Interestingly, although IL-6 is a well-established driver of RA pathogenesis, one study found that TNF-α siRNA and IL-1β siRNA produced more pronounced therapeutic effects in collagen-induced arthritis (CIA) mice compared to IL-6 siRNA, underscoring the importance of target selection and disease context (44).

Beyond inflammation, siRNA strategies have also been explored in restoring bone homeostasis and cartilage protection, which represent crucial but often underexplored aspects of RA pathology. Since RA progression involves disruption of the balance between osteoblast-mediated bone formation and osteoclast-mediated bone resorption, targeting molecules in these pathways may provide novel therapeutic avenues (45, 46). Furthermore, cartilage damage—often irreversible in RA—can be mitigated by siRNAs that reduce proteolytic enzyme activity, such as matrix metalloproteinases (MMPs). For example, Tang et al. designed a co-delivery system of MMP-9 siRNA and methotrexate (MTX), which demonstrated synergistic effects in reducing inflammation and reversing cartilage destruction in animal models (18).

Despite the promise of siRNA-based therapies, several challenges hinder their clinical translation. Naked siRNAs are inherently unstable, prone to degradation by serum nucleases, exhibit poor cellular uptake, and may be rapidly cleared via renal filtration. Additional barriers include immune activation, off-target effects, and inefficient delivery to inflamed synovial tissue. To overcome these obstacles, the development of nanocarriers—such as lipid nanoparticles, polymeric nanoparticles, and inorganic nanomaterials—has emerged as a critical strategy. These delivery systems enhance siRNA stability, prolong circulation, improve synovial targeting, and facilitate cytoplasmic release, thereby enabling RNAi to fully realize its therapeutic potential in RA.

Taken together, RNAi represents a transformative approach to RA treatment by specifically silencing pathogenic genes at the mRNA level. While recent advances—including the approval of Patisiran—underscore the translational feasibility of RNAi, the integration of target optimization and nanocarrier-based delivery systems will be essential to advance siRNA therapies from preclinical models to routine clinical application in RA.

## 4 Advances in nano-siRNA therapy for rheumatoid arthritis

Adeno-associated virus (AAV) has long attracted attention as a safe and efficient vector for gene delivery (47, 48). However, its application in the treatment of rheumatoid arthritis (RA) is limited. Given the symmetric, multi-joint involvement characteristic of RA, intra-articular injection is unsuitable for systemic therapy, while systemic administration of AAV suffers from insufficient biodistribution, reducing its therapeutic efficacy. Consequently, AAV may not represent the optimal carrier for siRNA-based therapies in RA. Currently, the most widely used siRNA delivery platforms in clinical studies are modified lipid nanoparticles (LNPs) and N-acetylgalactosamine (GalNAc) conjugates (49). The first FDA- and EMA-approved RNAi therapeutic, Patisiran, employs an LNP delivery system. Although LNPs have demonstrated relatively high transfection efficiency, they still face challenges such as limited tissue penetration, suboptimal cellular uptake, poor ability to cross the blood–brain barrier (BBB), and the requirement for intravenous rather than subcutaneous administration (34). GalNAc conjugates, which bind to the asialoglycoprotein receptor in hepatocytes, markedly enhance siRNA uptake into the liver, but they fail to fundamentally improve siRNA stability or achieve targeted delivery to extrahepatic tissues. In addition, the therapeutic duration of siRNAs remains relatively short, and long-term safety continues to require further validation. These limitations underscore the need for novel delivery systems.

Nanomaterial-based delivery systems (**Table 1**) provide a promising alternative for siRNA therapy in RA, as they can overcome many drawbacks of traditional carriers, including poor aqueous solubility, low bioavailability, and nonspecific biodistribution (50–52). Multifunctional nanocarriers can encapsulate sufficient amounts of therapeutic siRNA, prolong circulation half-life, and achieve targeted delivery to inflamed joints. Importantly, such systems can enable stimuli-responsive release based on pathological microenvironmental cues (e.g., acidic pH, elevated reactive oxygen species [ROS], overexpressed proteases) or external triggers such as ultrasound, magnetic fields, or irradiation (53). In addition, theranostic nanocarriers may incorporate imaging agents, allowing real-time monitoring of biodistribution, accumulation, and therapeutic response.

**Table 1:** Recent nanoparticle-siRNA for treatment of RA.

Nanomaterials	siRNA	Targeting	Route of injection	Model	Reference
Chitosan	TNF-α	Macrophages	*i.p.*	CIA	(47)
Chitosan	TNF-α	Macrophages	*i.v.*	CIA	(49)
Chitosan	Notch1	Notch signaling pathway	*i.v.*	CIA	(50)
FA-PEG-chitosan-DEAE	TNFα	Macrophages	*i.v.*	CAIA	(51)
RGD-PLGA	STAT1	Antigen-presenting cells	*i.v.*	CIA	(53)
PLGA-DOTAP	TNF-α	Macrophages	*i.a.*	CAIA	(54)
FA-PEG-PLGA	Mcl-1	B cells	*i.v.*	AIA	(55)
PEI/SPIO	IL-2/15Rb	Macrophages	*i.v.*	AA	(57, 58)
PEGylated liposome	TNF-α	Macrophages	*i.v.*	CIA	(60)
FA-PEGylated liposome-calcium phosphate	p65	Notch signaling pathway	*i.v.*	CIA	(19)
Lipid-PLGA	TNF-α	Macrophages	*i.a.*	DPIA	(61)
Lipid-polymer	IL- 1b	Macrophages	*i.v.*	CAIA	(62)
Lipid-PEG-*b*-PLGA	Bruton’s tyrosine kinase	Macrophages/B cells	*i.v.*	CIA	(63)
RVG-9R peptide	TNF-α	Macrophages	*i.v.*	CAIA	(66)
p5RHH peptide	p65	Notch signaling pathway	*i.v.*	CAIA	(67)
mPEGPCL-CH2R4H2C	RelA	Notch signaling pathway	*i.v.*	CIA	(65)
PLGA-DOTAP/PAMAM	TNF-α	Macrophages			(64)
Fe-MOF	Kelch-like ECH-associated protein 1	Macrophages	*i.v.*	CIA	(68)
Mesoporous silica	Mcl-1	Macrophages	*i.v.*	AIA	(69)

**Figure 2:**
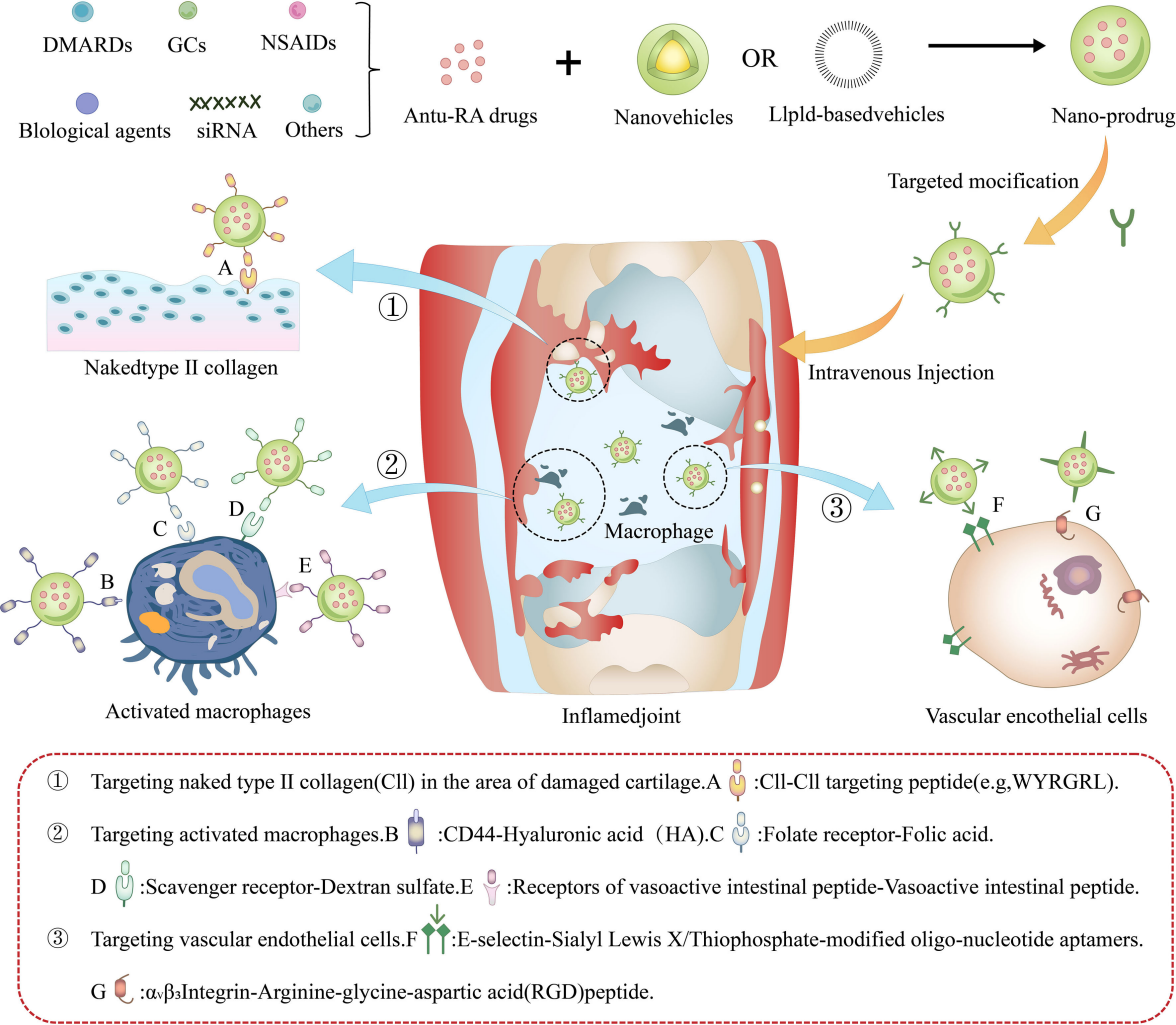
Schematic representation of multifunctional nanocarriers for active-targeted delivery of therapeutic agents in rheumatoid arthritis management.

For active targeting, nanocarriers can be functionalized with ligands that bind to overexpressed receptors in inflamed RA joints. For example, hyaluronic acid (HA) targets CD44, folic acid binds folate receptors, mannose ligands recognize macrophage receptors, and RGD peptides bind integrins (**Table 2**). These modifications significantly enhance cellular uptake in inflammatory synovial macrophages, fibroblast-like synoviocytes (FLS), and neovascular endothelial cells (**Figure 2**). Such multifunctional nanoplatforms have already shown great potential in cancer, cardiovascular, infectious, and inflammatory diseases, and are now being extended to autoimmune conditions such as RA. By improving delivery efficiency and enabling spatiotemporal control of gene silencing, nano-siRNA carriers provide a new direction for the precision treatment of RA.

**Table 2:** Nanomaterial-based targets for rheumatoid arthritis (RA) therapy.

Target	Ligand → receptor	Target cell/tissue	Therapeutic mechanism	Reference
Naked type II collagen (CII)	CII →CII targeting peptide	Damaged cartilage	Binds exposed collagen II in cartilage to enhance drug retention and cartilage protection	(54)
Activated macrophages	Hyaluronic acid (HA) → CD44	Synovial macrophages	Facilitates HA–CD44 interaction to promote macrophage uptake and reduce pro-inflammatory cytokine release	(55)
	Folic acid (FA) → Folate receptor	Synovial macrophages	Increases folate receptor–mediated uptake in activated macrophages	(56)
	Dextran sulfate → Scavenger receptor	Macrophages	Promotes receptor-mediated uptake and reduces inflammatory activity	(57)
	Vasoactive intestinal peptide (VIP) → VIP receptor	Macrophages	Suppresses inflammatory signaling via VIP receptor targeting	(58)
Vascular endothelial cells	Sialyl Lewis X/Thiophosphate-modified oligonucleotide aptamers → E-selectin	Vascular endothelial cells	Inhibits angiogenesis and reduces synovial vascularization	(59)
	RGD peptide (Arg–Gly–Asp) → Integrin α_v_β_3_	Vascular endothelial cells	Blocks integrin signaling, suppresses angiogenesis and pannus formation	(60)

### 4.1 Chitosan-based nanocarriers

Chitosan, a natural cationic polysaccharide derived from the deacetylation of chitin, has long been favored for biomedical applications owing to its excellent biocompatibility, biodegradability, low toxicity, and ease of chemical modification (61). These intrinsic properties have facilitated its widespread use in drug delivery, tissue engineering, and wound healing. Notably, its positive charge and strong electrostatic interactions with negatively charged nucleic acids make chitosan particularly attractive as a delivery vehicle for siRNA, enabling both efficient encapsulation and controlled release.

In an early landmark study, Kenneth et al. employed chitosan nanoparticles as carriers for TNF-α siRNA in collagen-induced arthritis (CIA) mouse models (62). *In vitro* experiments confirmed that chitosan/TNF-α siRNA complexes could specifically silence TNF-α expression in macrophages. Subsequent intraperitoneal administration in CIA mice demonstrated that even small amounts of siRNA encapsulated within chitosan nanoparticles were sufficient to significantly attenuate synovial inflammation, reduce pannus formation, and protect cartilage and subchondral bone. This pioneering work provided the first direct evidence supporting chitosan as a feasible nanocarrier for siRNA-based therapy in RA and laid the foundation for further translational research into RNAi therapeutics.

However, subsequent investigations revealed limitations in the use of chitosan with conventional siRNA. Owing to the short chain length and low charge density of TNF-α siRNA, electrostatic interactions alone were insufficient to consistently generate stable nanoparticles (63). To address this, Lee and colleagues proposed the concept of poly-siRNA, whereby chemically crosslinked and multimerized siRNA strands were complexed with thiolated glycol chitosan (tGC) (64). This strategy not only stabilized the nanocomplex but also improved intracellular delivery. In addition to TNF-α, the group developed tGC-based nanoparticles carrying siRNA against Notch1, a key receptor in the Notch signaling pathway implicated in cell proliferation, survival, and apoptosis (65). The resulting nanoparticles exhibited spherical morphology with an average diameter of ~260 nm, showed efficient uptake by macrophages, and effectively silenced Notch1 expression. In CIA models, *in vivo* imaging confirmed enhanced accumulation of siRNA at inflamed joints, which correlated with reduced synovial hyperplasia, suppressed inflammatory responses, and cartilage preservation.

With the advancement of nanotechnology, surface modifications of chitosan carriers have further expanded their therapeutic potential. Qin Shi’s team developed a folic acid (FA), polyethylene glycol (PEG), and diethylaminoethyl (DEAE)-modified chitosan nanocarrier (66). The FA moiety enabled active targeting of folate receptors on macrophages, thereby improving cellular uptake. When loaded with TNF-α siRNA and administered in collagen antibody-induced arthritis (CAIA) models, FA-PEG-DEAE/siRNA nanoparticles exhibited minimal cytotoxicity, robust targeting capability, and significant therapeutic efficacy. Specifically, treatment reduced arthritis scores, decreased TNF-α levels in synovial fluid, lowered pro-inflammatory cytokine release, and effectively protected cartilage and bone from destruction compared with control formulations.

Collectively, these findings underscore chitosan as a versatile and promising platform for siRNA delivery in RA. Its abundant natural availability, excellent safety profile, and amenability to functional modifications provide substantial advantages for clinical translation. Future research should focus on optimizing formulation stability, enhancing endosomal escape, and improving tissue-specific targeting to maximize therapeutic benefits. The demonstrated efficacy of chitosan-based nanocarriers highlights their potential not only for RA but also for broader applications in autoimmune and inflammatory disorders.

### 4.2 PLGA-based nanocarriers

Poly (lactic-co-glycolic acid) (PLGA) is a classical biodegradable polymer approved by the FDA for a variety of clinical applications. Combining the advantages of polylactic acid (PLA) and polyglycolic acid (PGA), PLGA has been widely employed in biomedical engineering, including surgical sutures, tissue repair scaffolds, and controlled-release drug formulations. Owing to its excellent biocompatibility and biodegradability, PLGA undergoes hydrolytic degradation into lactic acid and glycolic acid, which are readily metabolized via the Krebs cycle without causing significant inflammatory responses in surrounding tissues (67). These favorable characteristics have positioned PLGA as a promising nanocarrier for nucleic acid delivery, including siRNA-based therapies.

Inspired by earlier applications in oncology, Robert et al. designed an RGD-functionalized PLGA nanoparticle (RGD-PLGA) for siRNA delivery in RA (68). In this system, STAT1, a key transcription factor in interferon-γ (IFN-γ) signaling in antigen-presenting cells, was selected as the therapeutic target. *In vivo* imaging confirmed that RGD-modified PLGA nanoparticles significantly enhanced accumulation within inflamed joints. Treatment in experimental arthritis models revealed that RGD-PLGA/siSTAT1 nanoparticles effectively suppressed synovial inflammation, attenuated disease activity, and conferred superior cartilage protection compared with non-targeted controls. This work provided strong preclinical evidence that PLGA-based nanocarriers can achieve both targeted delivery and therapeutic efficacy in RA.

Despite its advantages, unmodified PLGA exhibits suboptimal transfection efficiency for siRNA delivery due to limited endosomal escape and inefficient cytoplasmic release. To overcome these barriers, Bernard et al. incorporated the cationic lipid dioleoyltrimethylammonium propane (DOTAP) into PLGA nanoparticles (69). The DOTAP-modified PLGA/siTNF-α formulation demonstrated improved cellular uptake and enhanced gene silencing efficiency, achieving significant anti-inflammatory effects in collagen antibody-induced arthritis (CAIA) mouse models. These findings suggested that cationic lipid functionalization can substantially enhance the performance of PLGA carriers.

More recently, Sun and colleagues reported an advanced multifunctional polymeric nanoplatform designed for targeted and stimuli-responsive siRNA release (70). The nanoparticles consisted of a pH-sensitive polymer (PK3) to facilitate controlled intracellular release, folic acid–polyethylene glycol–PLGA (FA-PEG-PLGA) as a targeting ligand, and DOTAP/siRNA as the therapeutic core. This platform demonstrated folate receptor–mediated uptake by macrophages and fibroblast-like synoviocytes, while the acidic microenvironment of inflamed synovium triggered specific siRNA release. In animal models, this smart FA-PEG-PLGA/siRNA nanocomplex effectively reduced synovial inflammation, suppressed pro-inflammatory cytokine production, and preserved joint integrity.

Taken together, these studies underscore the versatility of PLGA as a siRNA carrier in RA therapy. With appropriate surface functionalization—such as RGD peptides for angiogenesis targeting, DOTAP for enhanced transfection, or FA-PEG conjugates for macrophage-specific delivery—PLGA nanocarriers can integrate targeting, controlled release, and therapeutic efficacy. The modular nature of PLGA-based systems provides a robust platform for the development of next-generation RNAi therapeutics for autoimmune and inflammatory disorders.

### 4.3 Polyethylenimine-based siRNA delivery

Polyethylenimine (PEI) is one of the most extensively studied cationic polymers for nucleic acid delivery. As early as 2012, Charlie et al. reported a detailed protocol on the use of PEI as an efficient transfection reagent (71). Under physiological conditions, approximately one-fifth of the nitrogen atoms in PEI are protonated, conferring a high charge density and strong buffering capacity. This property enables the so-called “proton sponge effect”, which facilitates endosomal escape and enhances the cytoplasmic release of siRNA nanoparticles.

Zhang and colleagues employed PEI as a delivery vehicle for IL-2/15Rβ siRNA in adjuvant-induced arthritis models (72). Using Cy5-labeled PEI/siRNA complexes, they confirmed selective accumulation in inflamed joints, suggesting favorable biodistribution. Therapeutically, weekly administration of PEI/siRNA significantly slowed disease progression compared with controls, with reductions in joint swelling, inflammatory scores, and histopathological damage. These findings demonstrated that PEI can provide an efficient delivery platform for siRNA-based interventions in RA.

Nevertheless, a major limitation of PEI lies in its high positive surface charge, which induces substantial cytotoxicity and restricts its clinical translation. To address this, structural modifications of PEI have been investigated. For example, Zhang’s group developed superparamagnetic iron oxide nanoparticle (SPIO)-modified PEI, loading it with IL-2/15Rβ siRNA to construct a magnetically responsive nanocarrier (73). The PEI-SPIO/siRNA complexes not only improved joint-specific accumulation through enhanced permeability and retention (EPR) effects but also benefited from external magnetic field guidance, thereby achieving higher local concentrations and superior therapeutic outcomes.

In addition, combinatorial strategies integrating PEI with other nanomaterials have been explored to balance efficacy and toxicity. Ji Sun Park et al. designed PLGA-PEI hybrid nanoparticles encapsulating COX-2 siRNA for RA therapy (74). *In vitro* studies on C28/I2 cells showed that PLGA-PEI/COX-2 siRNA effectively suppressed inflammatory responses and downregulated apoptosis-related factors. These results highlight the potential of hybrid nanocarriers to overcome the individual shortcomings of PEI and PLGA while leveraging their complementary strengths.

Taken together, PEI remains a powerful siRNA delivery system due to its strong binding capacity and endosomal escape capability. However, cytotoxicity remains a critical hurdle. Emerging strategies such as chemical modification, hybrid carrier design, and magnetically responsive systems provide promising avenues to improve safety while maintaining high transfection efficiency. Future research should focus on optimizing PEI-based platforms for *in vivo* stability, joint-specific targeting, and reduced off-target toxicity to enhance their translational potential in RA therapy.

### 4.4 Lipid-based nanoparticles

Lipid-based nanoparticles (LNPs), including liposomes and lipid–polymer hybrid systems, have emerged as one of the most versatile and clinically validated platforms for nucleic acid delivery. Liposomes are spherical vesicles with bilayer membranes of 5–7 nm thickness and diameters ranging from 25 to 500 nm. Their amphiphilic nature enables encapsulation of both hydrophilic and hydrophobic therapeutic molecules, thereby enhancing membrane permeability and cellular uptake. Due to their favorable biocompatibility, biodegradability, and ease of surface modification, liposomal carriers are increasingly applied in gene therapy to improve the targeted delivery of siRNA, minimize off-target distribution, and optimize pharmacokinetics.

Abdulaziz et al. developed an acid-sensitive lipid-based nanocomplex (AS-TNF-α-siRNA-SLN) for RA treatment (75). This system achieved siRNA encapsulation efficiencies exceeding 90% and exhibited pH-responsive release. In collagen-induced arthritis (CIA) mice, AS-TNF-α-siRNA-SLN significantly reduced paw swelling, protected cartilage from cytokine-mediated degradation, and effectively prevented bone erosion. These findings provide strong preclinical evidence supporting the translational potential of acid-sensitive lipid carriers in RA therapy. Similarly, Duan and Li reported a folate-modified liposomal nanoparticle designed to deliver siRNA targeting NF-κB pathway components (20). The folate (FA) modification enhanced accumulation within inflamed joints by exploiting folate receptor overexpression on activated macrophages and fibroblast-like synoviocytes, resulting in improved anti-inflammatory efficacy and favorable safety profiles.

Despite these promising outcomes, a major limitation of conventional liposomes is their instability in serum-containing environments, leading to premature degradation and reduced therapeutic efficiency. To overcome this, hybrid nanocarriers combining lipids with polymers have been developed. These lipid–polymer hybrid nanoparticles harness the structural stability of polymers and the high encapsulation efficiency of lipids, thereby achieving improved circulation stability and intracellular delivery. For example, Jansende et al. employed liposome-PLGA hybrid nanoparticles to deliver TNF-α siRNA, demonstrating enhanced intracellular transport and reduced siRNA degradation prior to reaching target tissues (76).

Song et al. further advanced this concept by designing a lipid-polymer hybrid nanoparticle (FS14-NP) carrying siRNA against IL-1β (77). Upon intravenous administration, FS14-NP/siRNA rapidly accumulated in macrophages, effectively silenced IL-1β expression, and significantly alleviated ankle swelling, cartilage degradation, and bone erosion in CIA models. Beyond classical cytokine targets, Bruton’s tyrosine kinase (BTK), a critical regulator of B-cell and macrophage signaling, has recently emerged as a promising therapeutic target in RA. Zhao et al. encapsulated BTK siRNA in a liposome-PEG-b-PLGA hybrid system, which showed excellent therapeutic efficacy in suppressing synovial inflammation and joint destruction in experimental arthritis (78).

Collectively, these studies underscore the versatility and translational potential of lipid-based nanocarriers for siRNA delivery in RA. By incorporating functional modifications such as pH sensitivity, receptor-targeting ligands (e.g., folate, RGD peptides), and polymeric stabilizers, lipid-based platforms can achieve enhanced serum stability, targeted accumulation in inflamed joints, and controlled release of therapeutic siRNA. Nonetheless, further optimization is warranted to overcome residual limitations, including limited penetration into dense synovial pannus, potential immunogenicity, and scale-up challenges for clinical application.

### 4.5 Non-mainstream nanocarriers

In addition to lipid-, polymer-, and inorganic-based nanocarriers, several non-mainstream nanomaterials have been investigated for siRNA delivery in RA therapy (**Table 3**). Poly(amidoamine) (PAMAM) dendrimers represent one of the earliest explored cationic polymers, with abundant interior amine groups that electrostatically bind and protect siRNA from nuclease degradation during systemic circulation. Importantly, PAMAM nanoparticles demonstrated preferential accumulation in inflamed joints, thereby minimizing systemic off-target effects and enhancing therapeutic efficacy in experimental arthritis models (79). More recently, Takanori et al. developed an arginine-histidine-cysteine (RHC) oligopeptide-modified polymeric nanocarrier, which exhibited improved cellular uptake and efficient siRNA delivery, achieving significant anti-arthritic effects in collagen-induced arthritis (CIA) mice (80). In parallel, self-assembling short peptides have been reported as versatile siRNA carriers (81, 82). Their unique sequence motifs enable spontaneous formation of nanostructures that are readily internalized by macrophages, where intracellular enzymatic cleavage facilitates controlled siRNA release. This strategy enhances the specificity of gene silencing and reduces systemic toxicity, thereby offering an attractive alternative platform for targeted RNAi therapy in RA.

**Table 3:** Comparison of different nanocarrier systems for siRNA delivery in rheumatoid arthritis.

Nanocarrier	Loading efficiency	Biocompatibility/cytotoxicity	Targeting mechanism	Advantages	Limitations/challenges	Clinical readiness
Chitosan	Moderate (40–70%) via electrostatic interaction with siRNA	Excellent; Biodegradable and non-toxic	Passive uptake by macrophages; Modifiable with FA/HA for active targeting	Easy modification; Natural origin; Low cost	Limited endosomal escape; PH-sensitive stability	High – validated in multiple preclinical RA models
PLGA	Moderate to high (50–85%) depending on formulation	Excellent; FDA-approved, low immunogenicity	RGD/FA-mediated targeting; EPR-based accumulation	Controlled release; Scalable manufacturing	Limited endosomal escape; Hydrophobic degradation products	Very high – clinically used
PEI	High (>90%) due to strong electrostatic binding	Variable; High MW forms can be cytotoxic	Non-specific uptake; Magnetically guided (SPIO-PEI)	Strong condensation; Efficient endosomal escape	Cytotoxicity; Aggregation; Sterilization issues	Medium – modified low-MW forms under study
LNPs	High (80–95%)	Excellent when neutral; Cationic lipids may activate complement	Passive EPR; FA, RGD, HA-mediated active targeting	Clinically validated; Scalable and versatile	Stability; Rapid clearance; Limited deep penetration	Very high – FDA-approved RNAi drugs use LNPs
MOFs	High (>85%) from porous structure	Moderate; Fe-MOF biocompatible	CD44-mediated macrophage targeting (HA coating)	High loading; ROS/pH-responsive release	Metal ion toxicity; Scale-up challenges	Early preclinical – dual-drug co-delivery potential
Peptide/Dendrimer	Variable (40–90%) by charge density	Good post-PEGylation; Low toxicity	Receptor-mediated uptake (RGD, histidine/arginine motifs)	Tunable properties; Deep penetration	Complex synthesis; Possible immune activation	Moderate – early-phase clinical exploration

In addition, a modified iron-based metal-organic framework (Fe-MOF) system was engineered to co-deliver emodin (EM) and siKEAP1, with hyaluronic acid (HA) surface modification to enhance CD44-mediated uptake by pro-inflammatory M1 macrophages. This dual-delivery platform effectively reduced inflammatory cytokine secretion and reactive oxygen species (ROS) while promoting M2 macrophage polarization, ultimately alleviating joint pathology in collagen-induced arthritis (CIA) mice with an excellent biosafety profile (83). In parallel, mesoporous silica hybrid nanoparticles (PFHNs/TM) were designed by integrating photosensitizer PCPDTBT with tirapazamine (TPZ) and siMcl-1, and further functionalized with polyethyleneimine-folic acid (PEI-FA) (84). This multifunctional platform provided pH-responsive release and folate receptor-mediated targeting of activated macrophages, enabling a synergistic therapeutic strategy that combined RNAi, hypoxia-activated chemotherapy, and near-infrared (NIR)-induced phototherapy. Both *in vitro* and *in vivo* studies confirmed significant suppression of macrophage-driven inflammation and joint damage.

## 5 Conclusion and future perspectives

The treatment of rheumatoid arthritis (RA) remains a major challenge due to its complex and multifactorial pathogenesis, involving aberrant activation of immune cells, sustained pro-inflammatory cytokine networks, and dysregulated signaling pathways. Although conventional therapies such as methotrexate (MTX), nonsteroidal anti-inflammatory drugs (NSAIDs), and biologics can alleviate symptoms and slow disease progression to some extent, their efficacy is limited and often accompanied by resistance and adverse effects. Thus, the development of safer and more precise therapeutic strategies remains an urgent priority.

RNA interference (RNAi) has emerged as a promising molecular approach for targeted therapy in clinics. Small interfering RNAs (siRNAs), by specifically silencing pathogenic genes, exhibit high precision and selectivity, aligning with the principles of modern precision medicine. Selected siRNA formulations in the clinic (approved/late-stage) were shown in **Table 4**. Preclinical studies have demonstrated the therapeutic potential of siRNAs in suppressing critical cytokines such as TNF-α, IL-1β, IL-17, and VEGFA, as well as key signaling pathways including NF-κB, JAK/STAT, and MAPK. However, clinical translation of siRNA is hampered by inherent limitations, including instability, rapid nuclease degradation, low cellular uptake, and suboptimal biodistribution. Therefore, the design of efficient and safe delivery systems remains the critical bottleneck for RNAi-based therapeutics.

**Table 4:** Selected siRNA formulations in the clinic (approved/late-stage), by platform.

Platform	Example siRNA	Target/indication	Delivery modality	Mechanism of action	Challenges	Status
LNP	Patisiran (Onpattro)	TTR/hATTR amyloidosis	IV lipid nanoparticle	Hepatic delivery via ApoE-mediated uptake by hepatocytes;siRNA-induced RNAi silencing of transthyretin	Risk of infusion-related reactions (IRRs) requiring premedication; LNP-based delivery leads to liver and MPS (mononuclear phagocyte system) accumulation;	FDA/EMA approved (2018)
GalNAc	Vutrisiran (Amvuttra)	TTR/hATTR amyloidosis	SC GalNAc–siRNA	Tri-antennary GalNAc binds ASGPR on hepatocytes, enabling receptor-mediated internalization and durable TTR knockdown	Potential for cumulative off-target gene silencing with extended therapy; Requires life-long administration because disease is not reversed	FDA 2022
GalNAc	Inclisiran (Leqvio)	PCSK9/Hypercholesterolemia	SC GalNAc–siRNA	ASGPR-mediated hepatocyte targeting, RNAi-mediated PCSK9 suppression to lower LDL-C	Delayed onset of effect despite twice-yearly dosing; Requires careful monitoring in patients with renal impairment;	EU 2020; US 2021
GalNAc	Givosiran (Givlaari)	ALAS1/Acute hepatic porphyria	SC GalNAc–siRNA	Hepatocyte-targeted RNAi inhibition of ALAS1, reducing neurotoxic porphyrin intermediates	Risk of hepatic toxicity, including elevated ALT/AST; Possible worsening of renal function over long-term treatment;	FDA/EMA approved (2019)
GalNAc	Lumasiran (Oxlumo)	HAO1/Primary hyperoxaluria type 1	SC GalNAc–siRNA	Hepatocyte RNAi knockdown of glycolate oxidase to reduce oxalate production	Long-term RNAi suppression of glycolate oxidase may affect metabolic balance; Limited tissue targeting beyond the liver due to GalNAc system;	FDA/EMA approved (2020)
GalNAc	Nedosiran (Rivfloza)	LDHA/Primary hyperoxaluria	SC GalNAc–siRNA	Selective RNAi silencing of LDHA in liver to control oxalate generation	Variable treatment response associated with hepatic glycolate pathway differences; Long-term renal safety and metabolic effects require further study;	FDA approved (2023)

In recent years, the application of nanocarriers has provided new opportunities to overcome these challenges. Lipid nanoparticles (LNPs), poly(lactic-co-glycolic acid) (PLGA), polyethyleneimine (PEI), and chitosan have been widely investigated and validated as siRNA delivery vehicles for RA therapy. Moreover, emerging platforms such as metal-organic frameworks (MOFs), mesoporous silica nanoparticles, self-assembling peptides, and dendrimers have shown advantages in enhancing siRNA stability, prolonging circulation time, improving site-specific targeting, and achieving stimuli-responsive release. Particularly, intelligent nanoplatforms responsive to pathological features of the RA synovial microenvironment (e.g., acidic pH, elevated ROS, protease overexpression), as well as actively targeted systems modified with ligands such as folic acid, hyaluronic acid, or RGD peptides, have demonstrated significant potential for precise siRNA delivery.

Although current nanocarrier platforms for siRNA delivery—including lipid-based nanoparticles, polymeric micelles, dendrimers, and inorganic nanomaterials — demonstrate promising anti-inflammatory efficacy in RA models, each system exhibits important translational limitations that require critical evaluation. A major challenge is systemic stability: many polymeric and lipid-based vectors undergo opsonization and protein corona formation in circulation, accelerating their clearance by the mononuclear phagocyte system (MPS) and reducing effective half-life (85, 86). Efficient endosomal escape remains another key bottleneck. Cationic polymers such as PEI promote escape through the proton sponge effect but are associated with elevated cytotoxicity, whereas neutral or zwitterionic liposomal systems tend to remain entrapped within endolysosomes, limiting cytosolic siRNA release (87). Off-target biodistribution—particularly hepatic and splenic accumulation—has also been widely reported for both lipid nanoparticles and inorganic nanocarriers due to size- and charge-dependent sequestration (88). Comparatively, liposomes offer excellent biocompatibility but generally suffer from rapid systemic clearance without PEGylation; polymeric nanoparticles (e.g., PLGA, PU, PEG-PLys) provide better structural tunability and sustained release but face hurdles related to protein adsorption, batch consistency, and potential immunogenicity of cationic residues; inorganic nanocarriers such as gold nanoparticles, mesoporous silica, and MOFs offer high loading capacity yet raise concerns regarding long-term biodegradability and tissue retention. Together, these constraints underscore that despite clear advantages in stability, targeted delivery, and intracellular trafficking, the clinical translation of siRNA nanocarriers remains limited by systemic instability, endosomal entrapment, off-target effects, scale-up challenges, and incomplete long-term safety profiles. Future advances—including surface engineering, biomimetic coatings, and multi-responsive architectures—will be essential to push siRNA nanomedicine closer to clinical application in RA.

Several translational challenges remain before these systems can achieve clinical applicability (89). First, regulatory obstacles remain a major barrier. Both the U.S. FDA and EMA require siRNA nanoformulations to meet stringent standards regarding manufacturing reproducibility, nanoparticle size uniformity, sterility, endotoxin levels, and batch-to-batch stability (90). Many nanocarriers used in preclinical studies—such as polymeric micelles, dendrimers, inorganic nanoparticles, and hybrid systems—lack GMP-ready synthesis routes, making scale-up difficult (91). Additionally, regulatory agencies increasingly emphasize comprehensive nanocarrier characterization, including protein corona profiling, complement activation testing, long-term biodegradation behavior, and immunogenicity assessments (92), which remain under-reported in current RA nanomedicine research.

The other one is the formation of a protein corona upon systemic administration. Once exposed to biological fluids, nanoparticle surfaces rapidly adsorb plasma proteins such as albumin, apolipoproteins, and complement components (93, 94). This dynamic corona alters the physicochemical properties of the nanocarriers—affecting particle size, surface charge, and aggregation behavior—and consequently influences biodistribution, cellular uptake, and immune recognition (95, 96). In some cases, the corona may mask targeting ligands or trigger rapid clearance by the mononuclear phagocyte system (MPS), reducing therapeutic efficacy (97). Understanding and controlling protein corona composition through surface engineering (e.g., PEGylation, zwitterionic coatings, or biomimetic membranes) are therefore essential steps toward improving *in vivo* stability and reproducibility (98, 99).

Third, potential toxicity issues must be systematically evaluated prior to clinical application. Complement activation and the potential for immunotoxicity is a key concern. Positively charged or hydrophobic nanomaterials can activate the complement cascade via the classical or alternative pathways, leading to opsonization, cytokine release, or even hypersensitivity reactions (100, 101). Such immune activation not only compromises biocompatibility but may also exacerbate inflammation in the RA microenvironment (102). Furthermore, many nanocarriers accumulate in the mononuclear phagocyte system (MPS), warranting long-term histopathological studies of liver, spleen, and lymph nodes (103, 104). Without comprehensive toxicological data, regulatory approval for chronic autoimmune diseases remains challenging.

Fourth, dosage considerations are critical for clinical success. Optimizing the pharmacokinetic–pharmacodynamic (PK–PD) relationship is essential because siRNA degradation, rapid renal clearance, and endosomal entrapment can reduce therapeutic efficacy. Pharmacokinetic properties—such as circulation half-life, biodistribution, and metabolic clearance—directly influence pharmacodynamic outcomes including target gene silencing efficiency and duration of anti-inflammatory effects. For instance, nanocarriers with prolonged systemic retention and controlled siRNA release profiles can maintain sustained therapeutic concentrations at inflamed joints while minimizing off-target exposure (10, 105). Conversely, rapid clearance or premature siRNA degradation may result in suboptimal pharmacodynamic responses despite high *in vitro* delivery efficiency. Such data are largely absent from current RA-related siRNA studies.

Fifth, although clinical development of siRNA therapeutics has accelerated, clinical trials directly targeting RA remain limited. Several siRNA drugs—such as patisiran, givosiran, inclisiran, and vutrisiran—have gained FDA approval for other diseases, (**Table 4**)demonstrating the clinical feasibility of RNAi therapeutics (34, 106–108). These first-in-class examples provide regulatory precedents for formulation design, lipid nanoparticle (LNP) or GalNAc conjugate platforms, and validated safety profiles (109, 110). However, no siRNA nanomedicine has yet progressed to advanced clinical trials for RA. Existing early-stage studies primarily address inflammatory diseases or fibrotic disorders rather than autoimmune arthritis, highlighting an unmet translational gap.

Sixth, the substantial heterogeneity of the synovial microenvironment across different stages of RA is another important underappreciated barrier to clinical translation. Early RA is characterized by highly vascularized synovial tissue, abundant infiltrating immune cells, and a relatively loose extracellular matrix (ECM) architecture, which may facilitate deeper nanocarrier penetration and more efficient siRNA uptake by macrophages and fibroblast-like synoviocytes (111). In contrast, late-stage RA presents a markedly altered microenvironment: chronic inflammation leads to fibrotic pannus formation, increased ECM density, hypoxic niches, reduced vascular perfusion, and the emergence of distinct macrophage and fibroblast phenotypes (112, 113). These structural and cellular changes can impede nanoparticle diffusion, reduce endocytic activity, alter intracellular trafficking, and modify the pharmacodynamic response to siRNA (114). Moreover, cytokine gradients and receptor expression profiles (e.g., CD44, folate receptor β, integrins) evolve dynamically during disease progression (115, 116), potentially affecting the targeting efficiency of ligand-modified nanocarriers. Recognizing these temporal variations underscores the need for stage-adapted or microenvironment-responsive delivery systems, as a single nanocarrier design may not achieve uniform efficacy throughout the RA disease course.

Another major factor influencing the clinical translation of siRNA nanotherapeutics is the significant patient-to-patient variability in rheumatoid arthritis, which extends beyond disease stage and affects multiple biological determinants of therapeutic response. RA patients exhibit substantial differences in synovial cellular composition—such as variable proportions of macrophage subsets, fibroblast phenotypes, and infiltrating lymphocytes—which can alter nanoparticle uptake profiles and intracellular trafficking pathways (117). Genetic polymorphisms affecting cytokine production, RNAi pathway machinery, or endocytic receptors (e.g., CD44, folate receptor β, scavenger receptors) may further contribute to heterogeneity in target knockdown efficiency and ligand-mediated targeting specificity (118). Circulating and synovial cytokine signatures also differ markedly between individuals, shaping inflammatory gradients and modifying the physicochemical landscape encountered by nanocarriers (119). Moreover, inter-individual variability in pharmacokinetics—such as renal clearance rate, hepatic metabolism, complement activation tendency, and mononuclear phagocyte system (MPS) activity—may result in inconsistent biodistribution and exposure levels of siRNA formulations (120). Collectively, these factors underscore the need for personalized or stratified nanocarrier design, potentially incorporating biomarker-guided patient selection, adaptable targeting ligands, and microenvironment-responsive delivery strategies to achieve consistent therapeutic outcomes across heterogeneous RA populations.

Overall, siRNA-based nanomedicine represents a promising therapeutic paradigm for rheumatoid arthritis; however, its clinical translation remains constrained by several important challenges. Although current nanocarrier platforms demonstrate strong gene-silencing efficiency and encouraging anti-inflammatory outcomes in preclinical models, their behavior in humans is far less predictable. Key obstacles—including systemic stability, protein corona formation, endosomal escape efficiency, batch-to-batch manufacturing consistency, and long-term biosafety—must be rigorously addressed before clinical application can be realized. In addition, the heterogeneous nature of RA and its fluctuating inflammatory microenvironment imply that a single siRNA target or delivery strategy may not be universally effective across patient populations. Regulatory considerations also pose significant barriers, as siRNA nanomedicines must meet strict requirements for GMP production, quality control, sterility, pharmacokinetics, immunogenicity, and degradation profiling.
